# Drug-Resistant *Mycobacterium tuberculosis* among New Tuberculosis Patients, Yangon, Myanmar

**DOI:** 10.3201/eid0902.020128

**Published:** 2003-02

**Authors:** Sabai Phyu, Ti Ti, Roland Jureen, Thandar Hmun, Hlaing Myint, Aye Htun, Harleen M.S. Grewal, Bjarne Bjorvatn

**Affiliations:** * University of Bergen, Bergen, Norway; † National TB Programme, Yangon, Myanmar

**To the Editor:** Spread of drug-resistant tuberculosis (TB) and disastrous rates of HIV-TB co-infection pose serious threats to TB-control programs around the world (*1*). The World Health Organization/International Union Against Tuberculosis and Lung Diseases urges all national TB programs to practice the Directly Observed Treatment-Short Course (DOTS) strategy as well as to closely monitor the patterns and trends of anti-TB drug resistance (*2*). Such data allow an assessment of the quality of TB control, help forecast future trends of drug-resistance, and serve as guidelines for suitable therapy.

In 1997 the national TB programs of Myanmar introduced DOTS in the capital city, Yangon, which has approximately 5 million inhabitants. All new case-patients in national TB program clinics are routinely treated with isoniazid, rifampicin, ethambutol, and pyrazinamide without drug susceptibility testing of *Mycobacterium tuberculosis.* However, isolates from previously treated patients are frequently tested for drug susceptibility, and treatment is guided by the results. Myanmar is one of the 22 countries that account for 80% of the world’s new TB cases (*3*), yet little is known about drug-resistant TB in that country. We report on the pattern of drug resistance to first-line anti-TB drugs among *M. tuberculosis* complex isolates from Zone 1 TB center in Yangon, which receives approximately 70% of the national TB programs’ TB cases in Yangon. Of the 864 patients who attended this center in July 2000, a total of 202 were diagnosed as having pulmonary TB on the basis of medical history, clinical signs, two smear-positive sputum samples, and chest x-ray, if necessary. Approximately half of these cases were new pulmonary TB patients, i.e. smear-positive patients who had never been treated previously. Sputum specimens from 72 consecutive, new pulmonary TB case-patients were injected on Ogawa medium according to standard procedure (*2*); samples from 68 patients (94%) were culture-positive. Isolates from 17 patients were lost for further study because of bacterial contamination and failure to grow on subculture. Thus, isolates from 51 patients were available for the current investigation. By using the AccuProbe *Mycobacterium tuberculosis* complex test (Gen-Probe, San Diego, CA), all isolates were found to belong to the *M. tuberculosis* complex. Testing of isolates for susceptibility to isoniazid, rifampicin, ethambutol, and streptomycin was performed by using the standard Mycobacteria Growth Indicator Tube manual system, as recommended by the manufacturer (Becton Dickinson, Sparks, MD). The Wayne assay (*4*), which measures the activity of pyrazinamidase, was used for pyrazinamide susceptibility testing. This assay was performed according to World Health Organization guidelines for speciation within the *M. tuberculosis* complex (*5*). Eighteen isolates (35%) were resistant to any one of the five anti-TB drugs. Thirteen isolates (26%) were resistant to isoniazid, nine isolates (18%) to streptomycin, four isolates (8%) to ethambutol, one isolate (2%) to rifampicin, and one isolate (2%) to pyrazinamide. Only one isolate (2.0%) was multidrug resistant (MDR)-*M. tuberculosis*, i.e., resistant to both isoniazid and rifampicin.

The World Health Organization/International Union Against Tuberculosis and Lung Diseases global survey in the year 2000 (*6*) showed that the prevalence of resistance to at least one anti-TB drug (isoniazid, rifampicin, ethambutol, and streptomycin) among new cases ranged from 1.7% to 36.9%. In our study, 33.3% of the isolates from new pulmonary TB patients were resistant to at least one of these drugs. The finding shows that a relatively high frequency of drug resistance exists among our patients. If pyrazinamide is included in the calculation, the proportion of drug resistance among our patients is 35.3%. In 1994, Ti et al. reported that MDR-TB represented 1.25% of the isolates from 400 patients with newly diagnosed pulmonary TB who attended the Zone 1 TB center (*7*). When one considers the corresponding figure of 2.0% in the current material, frequency of MDR-TB in Yangon does not seem to have changed dramatically during the period 1994–2000. MDR-TB among new patients appears to be less common in Yangon than in big cities in Thailand (4.2%) (*8*) and in China (4.5%) (*6*). However, a substantial number of our isolates (15.7%) were resistant to two or more anti-TB drugs, in most cases to both isoniazid and streptomycin (9.8%). In the 1994 report by Ti et al., mono-resistance to streptomycin (6.5%) or isoniazid (5.8%) predominated, and 2.0% of the isolates were resistant to both isoniazid and streptomycin (*7*). Our present results, therefore, indicate that drug resistance is an imminent threat to TB-control efforts in Yangon, although MDR-TB still seems to be relatively rare.

The low number of MDR cases in our study could partly be explained by demographic features of the studied population, which is composed predominately of people residing in satellite townships of Yangon. These townships usually attract young people who immigrate to Yangon from village areas. These immigrants are less likely to have previous exposure to TB than the permanent population since the prevalence of TB infection is lower in rural than in urban areas (*9*). Moreover, population densities of the satellite townships are 2- to 10-fold lower than in inner Yangon city (Myanmar Central Statistical Organization). The high number of drug-resistance cases among our patients with newly detected TB could be explained by an undisclosed past exposure to anti-TB drugs. The case detection rate reported by the Myanmar national TB programs is 48% (*3*), suggesting that many TB patients receive their treatment elsewhere. A World Health Organization report (*10*) indicates that >80% of the health-care expenditure in Myanmar and other Asian countries such as India, Vietnam, and Cambodia is spent in the private sector. In such countries, poor treatment practices in the private sector may lessen the impact of the DOTS implemented by national TB programs and contribute to a growing incidence of drug-resistant TB. This problem will undoubtedly be escalated by the availability of free anti-TB drugs. HIV-TB co-infection often results in increased frequency of adverse drug effects, which may reduce compliance and increase induction of drug resistance. Although the prevalence of HIV positivity among our patients is unknown, a preliminary study from Yangon shows that the prevalence of drug-resistant TB among HIV-seropositive and -seronegative patients is the same (pers. comm., Myanmar nation TB programs]. To our knowledge, this report is the first to describe drug-resistant patterns in *M. tuberculosis* isolates from Myanmar.
